# A mixed exercise training programme is feasible and safe and may improve quality of life and muscle strength in multiple myeloma survivors

**DOI:** 10.1186/1471-2407-13-31

**Published:** 2013-01-24

**Authors:** Lara Groeneveldt, Gill Mein, Rachel Garrod, Andrew P Jewell, Ken Van Someren, Richard Stephens, Shirley P D’Sa, Kwee L Yong

**Affiliations:** 1 Cancer Institute, University College London, 72 Huntley Street, WC1E 6DD, London, UK; 2 Department of Haematology, University College London Hospitals, London, UK; 3 Faculty of Health Care Sciences, St. George’s University of London, London, UK; 4 MRC Clinical Trials Unit, London, UK; 5 English Institute of Sport, London, UK

**Keywords:** Myeloma, Bone disease, Exercise, Fatigue

## Abstract

**Background:**

Exercise programmes are beneficial for cancer patients however evidence is limited in patients with multiple myeloma (MM), a cancer that is characterised by osteolytic bone disease, giving rise to high levels of bone morbidity including fractures and bone pain.

**Methods:**

We conducted a single arm phase 2 study of an exercise programme (EP) as rehabilitation for treated MM patients, to evaluate feasibility, effects on QOL and physiological parameters. Patients were given individualised programmes, comprising stretching, aerobic and resistance exercises, carried out under supervision for 3 months then at home for a further 3 months.

**Results:**

Study uptake was high, 60 of 75 (80%) patients approached consented to the study. Screen failures (11, due to fracture risk and disease relapse) and patient withdrawals (12) resulted in a final 37 patients enrolling on the programme. These 37 patients demonstrated high attendance rates in the supervised classes (87%), and high levels of adherence in home exercising (73%). Patients reported better QOL following the EP, with improvement in FACT-G and Fatigue scores over time from baseline (p<0.01 for both, one-way repeated measures ANOVA) to 6 months. Upper and lower limb strength also improved on the EP, from baseline to 6 months (p<0.01 for both). There were no adverse reactions.

**Conclusions:**

An EP in MM patients is feasible and safe, with high attendance and adherence. Benefits in QOL, fatigue and muscle strength await confirmation in randomized studies, prompting urgent evaluation of the benefits of EP in the rehabilitation of MM patients.

## Background

Multiple Myeloma (MM) is bone marrow cancer of plasma cells that affects 15–20 per 100,000 people in the Western world, with a peak incidence in the 7^th^ decade [1]. A unique and integral feature of this cancer is osteolytic bone destruction, that is present in up to 70% of patients at diagnosis [2]. Severe bone pain is a frequent presenting symptom, and is a hallmark of lytic bone lesions, which in many patients results in long bone and/or vertebral compression fractures. Vertebral fractures lead to spinal deformity and instability, while spinal surgery and fixation often leave patients with chronic pain and reduced flexibility and mobility. Most patients initially respond to treatment, which has traditionally comprised chemotherapy and steroids +/− high dose therapy (HDT) with autologous stem cell transplantation (ASCT) and enter a plateau phase (remission) lasting a median of 3 years, before inevitable disease relapse. Although cure is rare, new effective therapies are extending survival in this cancer [3]. Despite enjoying longer remissions, many patients continue to suffer with the sequelae of bone destruction: persistent deformities, chronic pain, reduced mobility and physical functioning, and fatigue. Together with persisting toxicities of therapy such as neuropathy and steroid induced myopathy, these prevent a return to good personal and socio-economic functionality and erode well-being and QOL [4-7].

The benefits of exercise in cancer patients both during and after treatment are now well established, with evidence supporting positive effects on cardiorespiratory fitness and other physiological functions, including muscle strength [8-10]. Additional benefits for psychological and emotional well-being, fatigue, anxiety, and depression, although present, are less well supported in terms of quantitative analysis. Most of the evidence derives from studies of aerobic exercise in patients with solid tumours (breast, prostate) while the literature on patients with haematological cancers is less advanced. Many studies in this area have focused on patients undergoing chemotherapy or haemopoietic stem cell transplantation, with the aim of maintaining functional capacity, body composition and body weight or muscle mass, all of which parameters are known to decrease following intensive chemotherapy +/− stem cell transplantation [11]. Many of these studies used low-intensity or mixed exercise [12,13] and few reported QOL benefits. A notable exception is the study by Courneya and colleagues, who examined the effect of a progressive aerobic training programme on global QOL and physical functioning in lymphoma patients, including some on chemotherapy [14]. These authors report significant improvements in patient reported and objective measures of physical functioning.

There have been few studies of exercise in patients with MM. Skeletal deformities and the risk of further fractures, persistent pain and muscle wasting all render these patients unattractive candidates for exercise intervention. One recent study [15] examined the feasibility of exercise during treatment in 14 MM patients undergoing chemotherapy and HDT. Despite the small sample size, the authors found an individually prescribed exercise programme (EP) to be feasible and effective in maintaining body weight during chemotherapy. The same group evaluated the effect of an exercise programme in MM patients receiving erythropoietin whilst undergoing chemotherapy and ASCT [16]. Patients were randomized to a home-based exercise programme or usual care. Results indicated a beneficial effect on stem cell harvesting and blood product support. Both studies involved patients undergoing active treatment, however one randomised study, that included some patients with myeloma, examined the benefits of an EP as rehabilitation following a stem cell transplant [17]. The authors found that a mixed EP improved physical functioning but was without effect on QOL measures. Finally, an observational study in MM patients reported that engaging in even moderate exercise was associated with higher QOL scores [18]. These preliminary reports suggest that MM patients may benefit from regular exercise, and that EPs may be feasible in this patient group.

To provide further information on the feasibility, safety and efficacy of EP as rehabilitation strategy, we have carried out a pilot study of a tailored EP in treated MM patients. The aim was to assess feasibility and acceptability of an EP, and to obtain an estimate of the effect size in patient-reported outcomes, in order to power a future randomised study.

## Methods

Institutional ethical approval was obtained from the joint University College London and University College London Hospitals (UCL/UCLH) committee on the ethics of human research, Ref 06/Q0502/42, hence the study has been performed in accordance with the ethical standards laid down in the 1964 Declaration of Helsinki. Eligible patients (in stable plateau phase following chemotherapy and either off treatment or on maintenance therapy) were recruited. Exclusion criteria included spinal instability, risk of fracture, erythropoietin treatment, unstable angina, or musculoskeletal disease limiting mobility.

### Patient recruitment and screening

Suitable patients were identified in multi-disciplinary team meetings, or from outpatient clinics, and interested patients were given a Patient Information Sheet (PIS) to take home. Following informed consent, patients had a skeletal survey, laboratory profile to confirm stable disease and ECG where appropriate. Plain radiographs were assessed for fracture risk in a multi-disciplinary team meeting attended by a musculoskeletal radiologist, clinical oncologist, myeloma specialists, physiotherapist and clinical nurse specialists. Patients considered to be at risk of fractures, e.g. with large lytic lesions of the long bones or extensive lytic disease in the pelvis, underwent cross-sectional imaging with CT or MRI and were referred for surgery and/or radiotherapy. Patients who passed screening underwent baseline assessments for all study outcomes prior to starting on the EP.

### Study design

This was a single arm pilot study aimed at recruiting 40 patients. The primary objective was to assess the feasibility (accrual rate, acceptability and adherence to the programme) and safety (adverse events) of the EP. Secondary objectives were to assess the effect of the programme on overall QOL including fatigue, cardiorespiratory fitness, body composition and muscle strength. All patients undertook exercise training 3 times per week for 6 months. For the first 3 months, one session per week was a group session in the outpatient gym (supervised by the study physiotherapist) while the other 2 were home-based. In the subsequent 3 months, exercised sessions were home-based, and subjects attended the gym just once a month. Home-based exercise was supported by regular telephone contact with the study physiotherapist, who assessed all logbooks and maintained the clinical report form for each patient.

### Exercise programme

Each patient was given a programme based on their cardiopulmonary fitness and exercise capacity; programmes comprised stretching and mobility exercises, followed by aerobic and resistance training. Each session comprised both aerobic and resistance exercise training, with the aim to improve both cardiorespiratory fitness and muscle function. Aerobic exercise consisted of walking or stationary cycling, starting at 15 minute bouts at an intensity of 50% of heart rate reserve (HRR). During the aerobic training sessions in the gym, patients used heart rate monitors to maintain the prescribed heart rate and therefore control exercise intensity. To support monitoring of correct exercise intensity at home, patients were asked to report their rating of perceived exertion (RPE) using the Borg Scale [19]. Patients were given scales to take home, instructed in their use, and advised to work to levels of exertion as determined under supervision. Gradual progression in the exercise training was achieved by alternately increasing exercise duration by 5 min and exercise intensity by 5% HRR every 4 weeks, resulting an exercise session of 30 minutes duration at an intensity of 60% HRR in the final 4 weeks of the programme. All exercise programmes were prescribed on an individual basis to ensure suitability and to promote adherence to the programme.

Resistance exercises were individually tailored, targeting the major muscle groups for upper and lower limbs. Weight-lifting equipment, elastic exercise bands of varying resistance and body-weight were used for strengthening. When using the elastic exercise bands, the patient was started on the lightest resistance (colour coded accordingly). Likewise with the weight-lifting equipment, a low weight was initially used. Patients performed sets of repetitions, starting at 3 sets of 10 repetitions. The repetitions were progressed to 3 sets of 15 when deemed appropriate by the physiotherapist. The resistance or weight was increased when the patient felt that 3 sets of 15 at the current weight or resistance was no longer challenging. The repetitions then started again at 3 sets of 10 at the new resistance level or weight. This was the format in which the strength training was progressed, with the Borg scale being used to guide the progression. Resistance exercises were not modified for patients with vertebral fractures that were stable, except where required, eg for spinal deformity or lower back pain. In these cases, the position for execution of an exercise was changed from standing to sitting, or vice versa.

Each patient was given a demonstration of the exercises by the physiotherapist, followed by a return demonstration by the patient. Progression was achieved by increasing the resistance or the number of repetitions performed on each exercise. Each patient was given a booklet illustrating the exercises, and a log book to record the frequency, intensity and duration of the exercises, as well as their RPE on the Borg scale. The log books were used to adjust the exercise programme, as well as to assess adherence.

### Study outcomes

Feasibility was assessed by the rate of uptake, the screen pass rate, and the percentage of patients completing the programme. Acceptability was assessed by attendance rate in the gym-based classes, and by adherence to the programme, as scored from the log-books that patients brought in at each gym class. Adherence to the programme, was scored from the log books as percentage of exercise sessions completed over this period. Safety was assessed by the rate of adverse reactions (AR), ie adverse events (AE) that were clinically judged to be at least possibly related to the intervention, eg. increased bone pain, fractures or falls. Concomitant medication such as analgesia regimens were recorded to monitor pain levels, as a surrogate marker for AE’s.

Patient-rated and objective outcomes were assessed at baseline, 4-weekly in the first 3 months, and at 6 months from the start of the programme. Cancer-specific QOL was assessed using the Functional Assessment of Cancer Therapy General Cancer Scale (FACT-G). Fatigue was assessed by the 13-item Fatigue Subscale of the FACIT measurement system [20]. Baseline values were assessed in comparison with a reference population [21]. An increase in score indicates better QOL (FACT-G) and less fatigue (FACIT-F). Clinically significant changes in scores, termed Minimally Important Differences (MID) have been defined for these scales [22]. The total FACT-G and Fatigue scores have MIDs of 3–7, and 3–4 points respectively. The Hospital Anxiety and Depression Scale (HADS) was used to assess anxiety and depression [23].

Body mass, standing height, body composition (whole body fat and lean tissue, assessed using Bioelectrical Impedance Analysis), resting blood pressure and heart rate were measured as part of baseline assessment of cardiorespiratory fitness. Aerobic fitness was assessed using an 8 minute submaximal single-stage treadmill walking test [18]. This test allows for the estimation of VO_2max_ without the need for gas analysis or maximal exertion on the part of the patient, and has been validated as a method for assessing aerobic power by comparison with direct (i.e. expired gas analysis) measurements of VO_2max_[24]. For those patients who were unable to perform this test (eg dependence on a walking aid, or being unable to walk at a minimum speed of 2 miles an hour), a submaximal bicycle ergometer (Tunturi E6) and T-WARE ® software (Tunturi Ltd, Turku, Finland) were used to estimate VO_2max_. The test involves a computer braked and progressed ergometer protocol with increase in 25w every 2 minutes with HR monitoring. No patients were able to sustain load increases to full VO_2max_ hence submaximal tests were used. Isometric hand grip force, measured in kilograms, was assessed using a hand-held dynamometer; the greatest force out of three measurements from the right and left arm was taken, and the mean of these was used. Knee extensor strength was measured using a leg press, using 10 repetitions maximum (10RM), and was defined as the maximum weight that can be lifted just 10 times.

### Focus groups

Three focus groups were held to explore patients’ views. Twelve patients were invited and five men and five women attended. Focus groups were organised when sufficient patients were at a similar phase in the EP (weeks 6–12). The focus groups were facilitated by a trained qualitative researcher whom the patients had not met. A phenomenological approach was used [25] during the focus groups to gain an understanding of how the exercise intervention had impacted upon patients’ lives. Patients were asked how they felt the exercise classes had affected their lifestyles. Focus groups were recorded and transcribed. A thematic analysis [26] was used to categorize recurrent and common themes from the data, using the software package NVIVO (QSR 2006).

### Analysis

All outcome measures were assessed for changes from baseline. Assessments were performed every 4 weeks in the first 3 months, to provide information on the timescale over which effects were seen. A final assessment was performed at 6 months from the start of the programme. Planned analyses were carried out on data at 3 and 6 months, in comparison with baseline. Analyses included all participants who started the EP, regardless of adherence or attendance. Changes are summarized descriptively and comparison with baseline was carried out using a paired *t*-test (GraphPad PRISM). In addition, repeated measures one-way ANOVA was used where appropriate to evaluate changes over time. A p value of ≤0.05 is regarded as significant.

## Results

### Study uptake, screening and progression through the study

**Figure 1:**
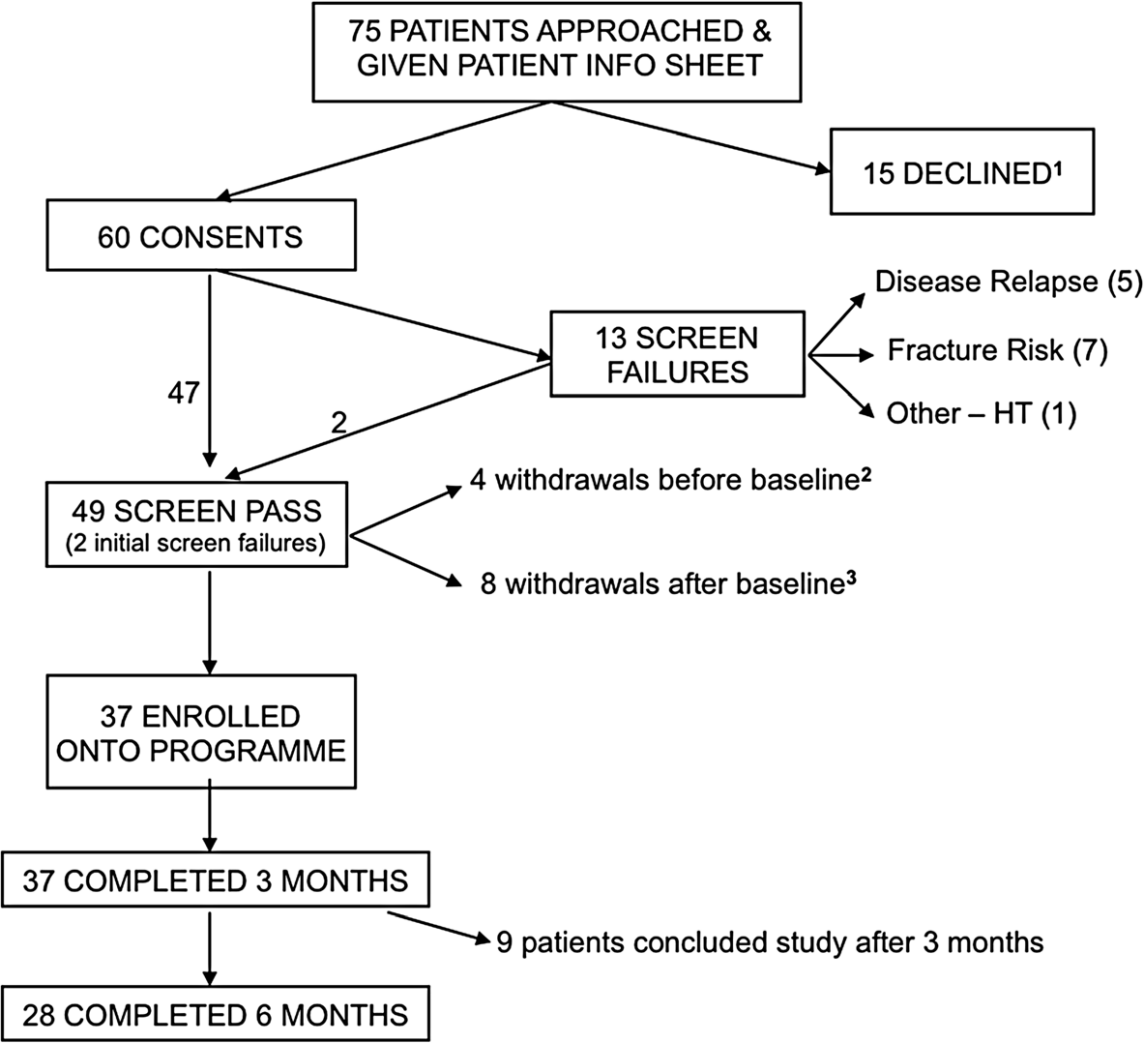
**Flow of patients through the study. **^1^ Seven patients declined because of travelling distance, 2 because they felt they were already undertaking sufficient exercise in their lifestyle, or at their local gym, 2 because they were not interested in the programme, and 4 because of family or work commitments. ^2^ Two patients withdrew because of travelling distance, and 2 because of family/work commitments. ^3^Three patients had disease progression, 4 withdrew because of family/work commitments, and 1 with depression.

Patients were recruited from October 2006 to December 2007. Of 75 eligible patients approached, 15 patients declined participation, largely due to personal or logistical reasons (Figure 1). Figure 1 shows the flow of patients through the study. There were 13 (21.6%) screen failures due to fracture risk (7), disease relapse (5) and hypertension (1). One patient had prophylactic surgery after screening and subsequently entered the programme, and another patient was enrolled a year later after repeat imaging confirmed he was no longer at risk. A total of 49 patients entered the study. Four patients withdrew before completing baseline screening tests. Of the 45 patients who completed baseline assessments, a further 8 patients withdrew prior to the start of the programme. All 37 patients who started on the programme completed 3 months, of these, only 28 were able to proceed to the second 3 months because of funding constraints but all of these completed the full 6 months.

### Patient characteristics, attendance and adherence, and safety

Of the 45 patients who completed baseline assessments, approximately half of the patients had significant bone disease with vertebral collapse and/or long bone fractures; 6 had undergone previous orthopaedic surgery and 11 received regular analgesia (Table 1). For the group as a whole, total FACT-G scores (83.0±12.7, mean±SD, range 57 – 105) were comparable to reference means from a normal healthy population in the US (80.1 ± 8.1) [21], while Fatigue scores (35.8 ± 11.6, range 13–52) were below the mean reference score (40.1 ± 10.4), ie patients experienced more fatigue. The 8 patients who dropped out after baseline assessments had similar FACT-G (80.5 ± 10.7), and Fatigue (31.8 ± 12.3) scores (both NS compared with the 37 patients who entered the programme). Of these 37 patients, 20 had significant bone disease, defined as lytic disease in more than one site, causing persistent pain, fractures and/or requiring surgery. A further 7 patients had moderate bone disease, defined as lytic disease in only 1 site, including fracture and /or surgery, but without persisting pain. Thus only 10 patients had asymptomatic or no bone disease.

**Table 1:** Patient characteristics

**CHARACTERISTIC**	
Age: Median (range)	61years (46–74 years)
Male	26 (58%)
Female	19 (42%)
Significant Bone disease^1^	23 (51%)
Time following completion of treatment	11months (median)
	(range 3–149 months)
Previous ASCT^2^	42 (93%)
Previous orthopaedic surgery	6 (13%)
Current therapies	
Maintenance treatment	9 (20%)
• Lenalidomide	1
• Interferon	2
• Thalidomide	6
Regular analgesia	11 (24%)

Details are given of patient characteristics for the 45 patients who completed baseline screening.

^1^ with one or more of the following: bone pain, vertebral collapse, fractures.

^2^ autologous stem cell transplantation.

Attendance in the exercise classes over the first 3 months of the study was high (87 ± 11%, mean±SD). Five patients failed to hand in their log books, but of the remaining 32 patients, adherence to the programme was 86 ±15 %. In the second 3 months, patients attended the outpatient gym only once every 4 weeks, and carried out the rest of the exercise sessions at home. Of the 28 patients who took part in the second 3 months, 20 handed in their log books; inspection of these revealed that adherence in the second 3 months was 73 ± 24%. All 28 patients attended for their 4-weekly gym sessions in the second 3 months (100% attendance). There were no adverse reactions, in particular there were no falls, or increases in bone pain in patients enrolled in the EP. On the other hand, many patients reduced their use of analgesia, and of 11 patients taking regular analgesics, 7 reduced or discontinued their medication, including 4 of the 6 patients on opioids.

### Patient reported outcomes

**Figure 2:**
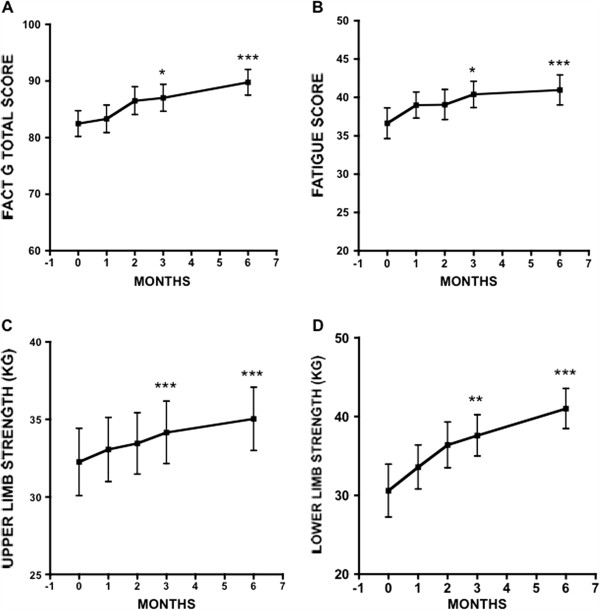
**Effect of exercise study on selected patient reported and physiological outcomes.** Changes in FACT-G (**A**), Fatigue (**B**), Upper limb strength (**C**) and Lower limb strength (**D**) over time. Mean ± SD of each measure.

Participation in the EP produced a marked improvement in patient-reported QOL, with significant increase in FACT-G scores (Table 2). For the 37 patients who completed 3 months on the EP, FACT-G scores improved from a baseline of 83.6 ± 13.1 (mean±SD) to 87.7 ± 13.4 at 3 months (p < 0.001, paired *t*-test). Importantly, 28 patients improved on their FACT-G scores at 3 months, and 22 of these achieved an MID (score change of > + 3). A one-way repeated measures ANOVA determined that FACT-G scores differed significantly between time points (F = 9.71, p < .001) for the 28 patients who completed 6 months. Post-hoc comparisons using the Bonferroni correction showed a general improvement over time, + 4.5 from baseline to 3 months (95% CI: -0.2 to 9.3, p = 0.062), + 7.3 from baseline to 6 months (95% CI 2.7 to 11.9, p = 0.001), and + 2.8 from 3 months to 6 months (95% CI: -0.6 to 6.1, p = 0.141) (Figure 2).

**Table 2:** Changes from baseline FACT-G and Fatigue scores for 37 patients who completed 3 months (top) and for 28 of these who completed 6 months (bottom)

	**BASELINE mean±SD (range)**	**3 MONTHS mean±SD (range)**	**CHANGE in mean**	***P***	**Number of patients who**		
					**Improved (MID)**	**Worsened**	**No Change**
**FACT-G**	83.6 ± 13.1	87.7 ± 13.4	4.1	<0.001	28	9	0
**n=37**	(62–105)	(53–108)			(22)		
**FATIGUE**	37.4 ± 10.4	40.5 ± 9.0	3.1	<0.01	25	9	3
**n=37**	(14–52)	(19–52)			(17)		
	**BASELINE Mean±SD (range)**	**6 MONTHS mean±SD (range)**	**CHANGE in mean**	***P***	**n**		
					**Improved (MID)**	**Worsened**	**No Change**
**FACT-G**	82.5 ± 12.1	89.8 ± 12.1	7.3	<0.001	23	3	2
**n=28**	(62–104)	(69–107)			(22)		
**FATIGUE**	36.6 ± 10.6	41 ± 10.3	4.4	<0.001	23	4	1
**n=28**	(14–50)	(16–52)			(18)		

Significance values for comparison of baseline scores with scores at 3 and 6 months are indicated (using paired *t*-test). Analysis of changes over time by one-way repeated ANOVA is given in the Results section.

Patients also reported less fatigue on the EP. This was reflected in a significant improvement in fatigue scores at 3 months, increasing from 37.4 ± 10.4 at baseline to 40.5 ± 9.0 (p < 0.01, *t*-test, Table 2). Seventeen of these patients achieved an MID (score change of > + 3). For the 28 patients who completed 6 months, fatigue scores differed significantly over time points (F = 7.08, p = 0.002), with a general improvement over time, +3.8 from baseline to 3 months (95% CI −0.1 to 7.6, p = 0.056), + 4.3 from baseline to 6 months (95% CI: 1.5 to 7.1, p = 0.001) and + 0.6 from 3 to 6 months (95% CI: -2.3 to 3.4, p = 1.0). These changes are illustrated in Figure 2.

For the HADS scores, 9 of the 37 patients who completed 3 months had a baseline anxiety score of ≥ 8 (borderline or case), in 4 of these, their scores had improved to 7 or less at 3 months. Six patients had a baseline depression score of ≥ 8, and 2 had improved to a score of 7 or less at 3 months. In the group that completed 6 months on the EP, anxiety scores did not change (7 had scores of ≥ 8), while 2 out of 6 patients with baseline depression scores of ≥ 8 had improved to 7 or less by 6 months. Table 3 gives the overall scores at each time point.

**Table 3:** HADS scores at baseline, 3 and 6 months

	**Normal**	**Borderline**	**Case**
Anxiety			
Baseline (n=37)	28	6	3
3 months (n=37)	30	5	2
6 months (n=28)	20	6	1
Depression			
Baseline (n=37)	31	4	2
3 months (n=37)	31	4	2
6 months (n=28)	23	4	1

The total number of patients in each group is also indicated. (Normal = score 0–7,

Borderline = score 8–10, Case = score 11–21).

### Physiological outcomes

Upper limb strength improved significantly over time points (F = 11.81, p < 0.001, one-way repeated measures ANOVA), + 1.9 from baseline to 3 months (95% CI: 0.6 to 3.2, p = 0.003), + 2.8 from baseline to 6 months (95% CI: 0.9 to 4.6, p = 0.002), and + 0.9 from 3 to 6 months (95% CI: -0.4 to 2.1, p = 0.277). Lower limb strength also improved significantly over time (F = 12.01, p < 0.001), + 7.0 from baseline to 3 months (95% CI: 1.2 to 12.9, p = 0.015), + 10.4 from baseline to 6 months (95% CI: 4.0 to 16.8, p = 0.001), and + 3.4 from 3 to 6 months (95% CI:- 0.9 to 7.7, p = 0.152). Figure 2 shows the time frame of changes from baseline to 6 months in these 28 patients. There was no significant change in aerobic capacity, as measured by VO2 max (F = 3.07, p = 0.057). Table 4 summarizes the data for the 28 patients who completed 6 months on the study.

**Table 4:** Changes in muscle strength, V02 max and fat-free mass index

**n=28**	**BASELINE (mean ± SD)**	**3 MONTHS (mean ± SD)**	**6 MONTHS (mean ± SD)**
**Upper Limb Strength**	32.3 ± 11.5	34.2 ± 10.7	35 ± 10.8
**(Kilograms)**			
**Lower Limb Strength**	30.6 ± 16.8	37.6 ± 13.1	41 ± 12.7
**(Kilograms)**			
**Vo2 Max**	27.8 ± 5	28.2 ± 4.5	28.1 ± 4.8
**(Ml/Kg/Min)**			
**Fat-Free Mass Index**	18 ± 2.3	18 ± 2.5	18 ± 2.5

Scores at baseline, 3 and 6 months are given for the 28 patients who completed 6 months on the programme.

### Focus groups findings

Patients invited to the focus groups had been on the programme for two to five months. Ten patients (5 men and 5 women) attended three focus groups (one male, one female and one mixed). Several themes were identified. One was the fear associated with the risk of bone damage. The diagnosis of MM was itself frightening and patients described how they were warned of the risk of bone fractures. Hence patients were unsure what exercise was safe, many were not exercising before the study. Patients described how their lives had been transformed by the exercise intervention. They appreciated the programmes were designed to suit individual needs, and felt secure when advised and supervised by a trained physiotherapist. A second theme was an increase in confidence. Patients felt the programme had empowered them, and improved their confidence in other areas of their lives. They reported new activities outside the home, including long walks or travelling abroad. Another theme was the support that the patients gained from contact with other MM sufferers. Observing how fellow sufferers coped gave patients hope and enabled them to talk about their future. Thus, the group exercise experience seemed to influence their perception of the future.

## Discussion

The principal finding of this study is that a tailored EP is safe and feasible in treated MM patients. Patient participating in the study demonstrated improvements in QOL measures, particularly fatigue, and in muscle strength, suggesting possible benefits of such an EP. Our study is the first to systematically explore the feasibility and benefits of a tailored EP in the rehabilitation of treated MM patients. The high rate of uptake (80% of eligible patients) compares favourably with a RCT in lymphoma patients (26%, ref.14) and attests to the keenness of these patients to engage in an EP, despite the perceived frailty of their bones. The attrition rate (24%) was similar to that reported for exercise programmes in other cancer patient populations [27,28], and compares favourably with one of the few reported studies in MM patients (42%, Coleman et al.). Importantly, all patients who started on the EP completed their planned 3, or 6 months. This, together with the high attendance rates in the gym classes (87% in first 3 months, 100% in second 3 months), indicates the acceptability of the EP. Adherence was only assessable in patients who returned their logbooks (71-86%), but levels were acceptable (72% and 86%), and many patients testified that they performed the EP at home, despite not filling in the log books. We also confirmed that a tailored mixed EP is safe, in that there were no AR’s.

In this era of MM therapy where new and effective treatments are increasing remission rates and extending survival [3], it is vital to focus on non-drug strategies that will help to maximise wellbeing and QOL for survivors. The inclusion of EP in rehabilitation is a novel approach because hitherto, few clinicians have advised their patients to engage in exercise, for fear of further bone damage. On the other hand, their bone pathology and skeletal complications mean that MM patients have much to gain from exercising. Exercise improves bone health, as shown by studies in women at risk of osteoporosis where weight bearing exercise increased bone density [29]. Resistance exercise, by improving muscle mass, improves strength and balance, reducing the rate of falls which is a major risk factor for fractures [30]. Previous studies in this patient group have excluded subjects with lytic bone disease, thus to the best of our knowledge, this is the first study to demonstrate that MM patients with significant bone disease are able to exercise safely. Our results will make an important contribution to the development of rehabilitation programmes for these patients.

Due to their bone disease and generally older age, (median age of MM survivors is 70 years) many MM patients may not be suited to even moderately intensive aerobic programmes, hence the inclusion of resistance exercises is an important feature of the EP. Resistance exercises can reduce fatigue, improve QOL and muscle strength, and produce longer term improvements compared with aerobic exercises [31]. Because of their bone disease, patients were given individually tailored programmes, and attended supervised weekly exercise sessions, factors that are likely to contribute to the safety of the EP. Testimonies from the focus groups indicated that patients found the supervised sessions reassuring, gaining confidence to undertake new physical activities. Because we found that some patients were unable to perform the single stage submaximal treadmill walking test, we used an alternative method of estimating VO_2max_; future studies should standardise the test for cardiorespiratory fitness. To improve on the logbook return rate, patients may be offered incentives, and given positive reinforcement in the form of follow-up telephone calls from the physiotherapist.

Our study was designed as a single arm pilot study, which clearly presents limitations when interpreting the results. In particular, because subjects are compared only to themselves previously, this design does not allow us to conclude that the improvements in patient reported and objective outcomes are necessarily due to the intervention. It is possible that patients would have experienced improvements in these parameters over time. A comparator group of patients, in a randomized study, is required to answer this question. Some insights however, may be derived from the focus groups. A focus group is a more natural situation than an interview as the participants share and compare experiences and opinions. The results thus provide a powerful insight into experiences, beliefs and attitudes [32,33]. Patients reported benefits from meeting other MM sufferers, such as increased confidence and hope for the future, and thus patients may gain from engaging in physical activity together. In an RCT of a group-based exercise programme in breast cancer patients Mutrie and co-workers concluded that some of the benefits observed derived from the group experience [34].

A potentially important finding is the improvement in fatigue levels following the EP. Because of the limitations of a single arm study, we cannot conclude that this is due to the EP, however, these findings warrant further investigation. Fatigue is a prominent symptom in cancer patients [35], and one of the widely reported benefits of exercise training is a reduction in fatigue, however not all studies have shown statistically significant effects [8,34,36,37], and much of the evidence derives from patients undergoing treatment. There is less information in patients who have completed therapy, however a single arm study reported that 32 cancer patients, after a 3-week programme of endurance and resistance exercise, had improved physical performance and reduced fatigue levels [38]. The mechanisms whereby exercise lessens fatigue are not completely understood, but may relate in part to improved sleep patterns [39,40]. Future work could explore this mechanism by including a measure of sleep. Reduced fatigue would particularly benefit this older, more frail cancer group with bone morbidity as it would lead to increased activity and functionality, with attendant benefits on wellbeing and social functioning.

## Conclusion

In conclusion, we demonstrate that a prescribed EP for treated MM patients is feasible, acceptable and safe. The findings in this single arm study await urgent confirmation in a randomised trial to evaluate the benefits of exercise intervention as rehabilitation in these patients. The longer term benefits of an EP, and the potential for a sustained lifestyle change also need to be explored. While the results of randomised trials are awaited, our observations suggest that physicians can recommend regular exercise to MM survivors, provided suitable screening measures are undertaken, and there is appropriate input from trained physiotherapists.

## Competing interest

The authors have no financial relationship with the sponsor of this study. We have full control of all primary data and will allow the journal to review such data if requested. The authors declare that they have no competing interests.

## Authors’ contributions

LG carried out the exercise programme, analysed the results and drafted the paper, GM carried out the focus groups and analysed the results, RG participated in the design and supervision of the study, APJ conceived of the study and participated in the design and supervision of the study, KVS participated in the design of the study, RS carried out statistical analysis, SDS participated in the design and coordination of the study, KLY conceived of the study, participated in the design and supervision of the study and wrote the paper. All authors read and approved the final manuscript.

## Pre-publication history

The pre-publication history for this paper can be accessed here:

http://www.biomedcentral.com/1471-2407/13/31/prepub
